# RNA-binding proteins signature is a favorable biomarker of prognosis, immunotherapy and chemotherapy response for cervical cancer

**DOI:** 10.1186/s12935-024-03257-w

**Published:** 2024-02-21

**Authors:** Xiaomei Chen, Xunhu Dong, Hong Li, Tingting Wu, Haoyin Liu, Jie Wu, Wei Ge, Lingji Hao, Zhe Zhang

**Affiliations:** 1 Nursing Department, Medical Centre Hospital of Qionglai City, Qionglai, 611530 Sichuan China; 2 Institute of Toxicology, School of Military Preventive Medicine, Third Military Medical University (Army Medical University), 30 Gaotanyan Street, Shapingba District, Chongqing, 400038 China; 3 Department of Obstetrics and Gynecology, Xinqiao Hospital, Third Military Medical University (Army Medical University), Chongqing, 400038 China; 4 Department of Obstetrics and Gynecology, Southwest Hospital, Third Military Medical University (Army Medical University), Chongqing, 400037 China

**Keywords:** RNA-binding proteins, CESC, Prognosis, Immunotherapy, Chemotherapy

## Abstract

Cervical squamous cell carcinoma and endocervical adenocarcinoma (CESC) still present a huge threaten to women’s health, especially the local advanced patients. Hence, developing more effectiveness prognostic signatures is urgently needed. This study constructed and verified a robust RNA-binding proteins (RBPs) related signature through a series of bioinformatics methods and explored the biological function of hub RBP in vitro experiments. As a result, the 10 RBPs signature was successfully established and could act as an independent prognostic biomarker in CESC patients, which displayed the highest sensitivity and specificity in prognosis prediction compared with other clinicopathological parameters. The risk model also presented good performance in risk stratification among CESC patients. Besides, a nomogram was constructed based on pathological stage and the risk signature and exhibited satisfactory accuracy in prognosis prediction. Functional enrichment indicated that the risk signature mainly participated in immune-related pathways and cancer-related pathways, and the infiltration level of immune cells and immune checkpoints showed a significantly higher degree in low-risk patients compared with high-risk patients. Notably, the 10 RBPs signature act as a novel biomarker in immunotherapy and chemotherapy response. In addition, PRPF40B was selected as hub RBP and its transcription and translation levels were obviously increased in CESC tissues, as well as Hela and Siha cells. Knockdown of PRPF40B inhibits the proliferation, migration and invasion of Hela and Siha cells in vitro. In conclusion, our research provides a noticeable strategy in prognostic prediction among CESC patients, which may illuminate the prospect of CESC patients’ clinical outcome.

**Supplementary Information:**

The online version contains supplementary material available at 10.1186/s12935-024-03257-w.

## Introduction

Cervical squamous cell carcinoma and endocervical adenocarcinoma (CESC) have been the fourth leading cause of morbidity and mortality among women cancers, with approximately 604,127 (3.1%) newly incidences and 341,831 (3.4%) newly death in 2020 [1]. CESC is curable in the early stage, but the locally advanced cases with or without distant metastasis usually present poor prognosis even though the combination therapeutic including surgical intervention, radiotherapy and chemotherapy have been implicated in these patients [2]. Recently, emerging prevention strategies have been launched into clinical practice, including human papillomavirus (HPV) vaccine and early cervical screening, after WHO called for elimination of CESC all over the world [3]. With the development of detection technology, increasingly advanced CESC has been diagnosed earlier and more accurately. Meanwhile, novel measures such as the target therapy and immunotherapy also have been applied [4, 5]. However, limited by tumor heterogeneity and economic factors, the above treatment measures are difficult to be effectively implemented, thus, CESC is still a major threaten to poor prognosis among females, especially in the developing countries [6, 7]. Nowadays, next-generation sequencing has been widely employed in various aspects of cancer research, and it is becoming an effective method to extensively screen biomarkers for prognosis evaluation and therapy target selection [8]. Comprehensively analysis of genome transcription and multi-omics analysis could select promising biomarkers in a less expensive way, which may alleviate the nonnegligible burden generated by HPV vaccination and cytological examination [9]. Therefore, discovering the novel biomarkers for CESC is urgently needed and could provide valuable information for further research.

RNA binding proteins (RBPs) are proteins that interact with many types of RNA. So far, more than 1500 RBPs have been found in the human genome [10]. RBPs participate in the post transcriptional regulation of RNA, determine the function of each RNA transcript in cells, and ensure cell homeostasis [11]. They establish highly dynamic interactions with other proteins and coding RNA and non-coding RNA to form functional units called ribonucleoprotein complexes, which have the functions of regulating RNA splicing, polyadenylation, stability, localization, translation and degradation [12]. In gynecological malignant tumors, more and more abnormally expressed RBPs have attracted people’s attention. They play an important role in the occurrence and development of tumors by influencing the proliferation, apoptosis, epithelial mesenchymal transition (EMT), invasion and metastasis, drug resistance and other processes of cancer cells [13]. In the future, they may be helpful for early diagnosis or serve as a target for the treatment of tumor recurrence, so as to improve the prognosis of patients and prolong their survival time [14]. In addition, RBPs may be used as diagnostic markers or potential therapeutic targets, and could participate in the proliferation, cell cycle, apoptosis, drug resistance and other related biological processes of CESC [15]. Therefore, a comprehensive analysis of the biological function and prognosis of RBPs will help to better understand the occurrence and development of CESC, and may reveal new targets for therapeutic application and provide further ideas and references for subsequent research.

Here, we firstly identified differentially expressed RBPs between paracancerous tissues and CESC, then constructed a RBPs’ signature based on this basis. A nomogram was also established to comprehensively investigate the clinical implication of RBPs’ signature. Furthermore, the mutational landscape, responses to immunotherapy and chemotherapy of different risk populations were explored through various bioinformatics methods and webtools. Finally, the biological function of PRPF40B was verified in vitro experiments. Our study mainly focused on the prognosis and functions of RBPs, hoping the results would shed light on the fog of RBPs in CESC, and provide useful information for clinicians.

## Results

### Workflow schedule of this study

**Fig. 1:**
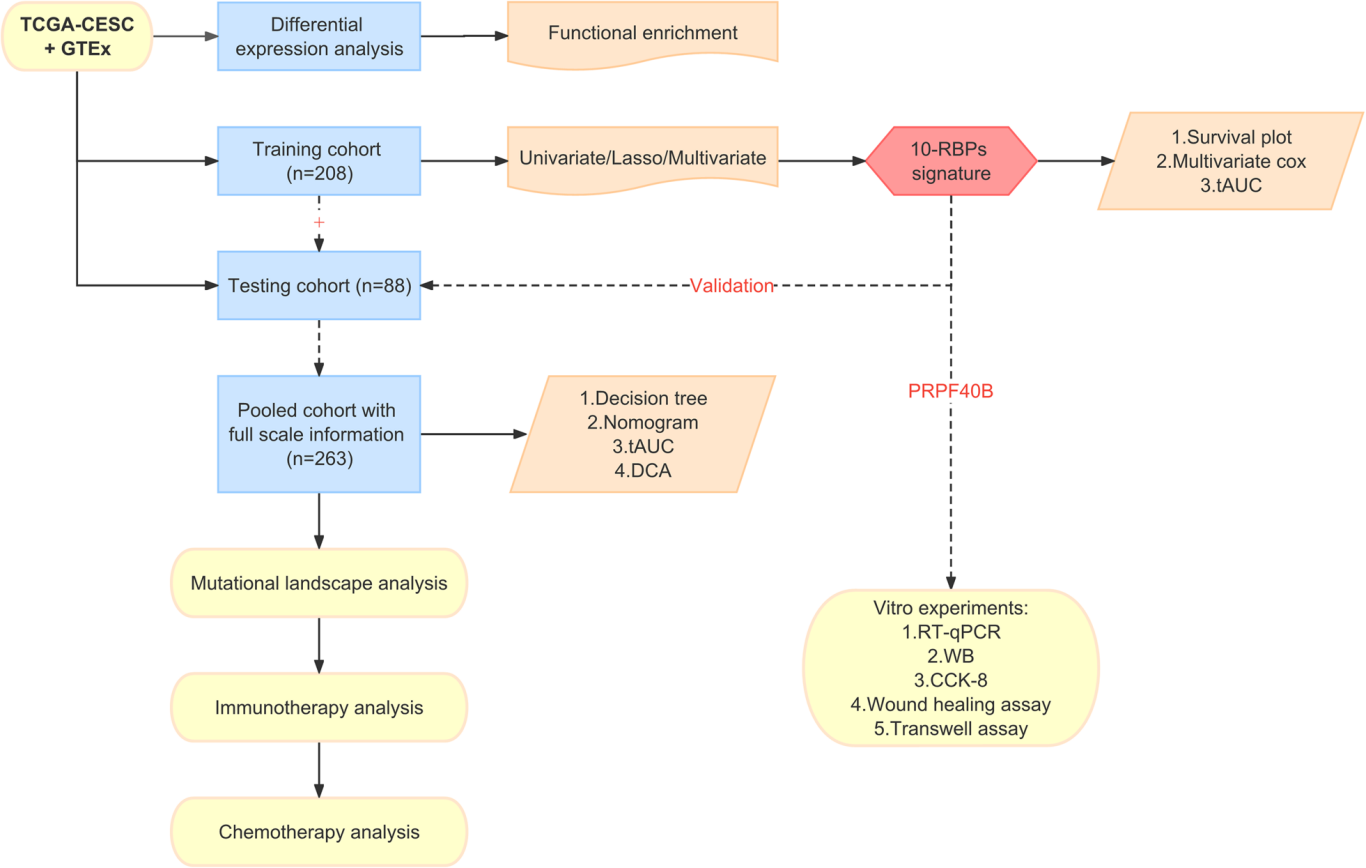
Schematic diagram of this research

The technology schedule of this study was presented in Fig. 1. Firstly, we explored the differentially expressed RBPs between paracancerous and CESC, and the corresponding functional enrichment analysis was also conducted. Secondly, the TCGA-CESC cohort was separated into a training cohort (208 patients) and a testing cohort (88 patients) according to the ratio of 7:3. Univariate Cox regression, lasso Cox regression and stepwise multivariate Cox regression were employed to select candidate hub RBPs and a 10-RBPs signature was finally constructed in the training cohort. The risk signature was evaluated through survival analysis, multivariate Cox analysis and tAUC in the training cohort and then validated in the testing cohort. Thirdly, the training cohort and testing cohort were integrated into a pooled cohort and the patients with full scale of information were retained for further analysis. The decision tree was established to optime the risk stratification of CESC patients and the nomogram was constructed to quantify the risk value of CESC patients. Then the nomogram was assessed through calibration curves, tAUC and DCA. Fourth, the study investigated the mutational landscape, effect on immunotherapy and chemotherapy response of the RBPs’ signature through multiple bioinformatics methods and online webtools. In addition, the expression of PRPF40B was detected in CESC cell lines and clinical tissues, respectively. Finally, the biological functions of PRPF40B were explored by CCK-8, wound healing and transwell assay in vitro experiments.

### Construction of 10-RBPs signature in CESC

**Fig. 2:**
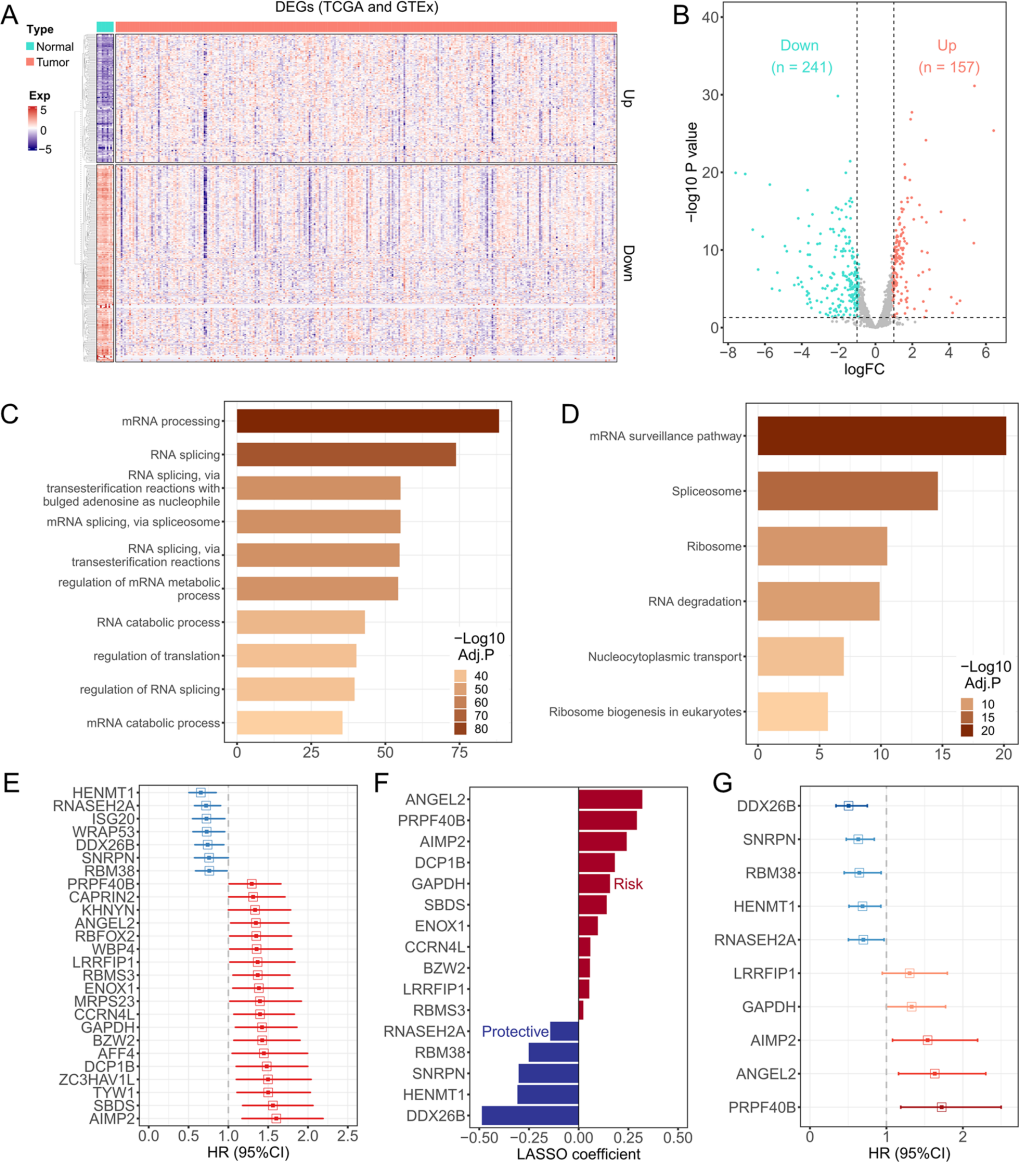
Construction the risk signature in training cohort. **A** Heatmap was drawn to show the DEGs between normal cervical tissues and CESC with the following criterion: |logFC|> 1 and adjust *P* value < 0.05; **B** A total of 398 DEGs were presented in volcano plot, up-regulated DEGs were displayed as red points and down-regulated DEGs were displayed as green points; **C** Top 10 enriched biological process of these 398 DEGs; **D** KEGG enrichment results of these 398 DEGs; **E** 26 DEGs showed statistical significance (*P* < 0.05) through univariate Cox regression, DEGs with HR < 1 were presented as blue bars and DEGs with HR > 1 were presented as red bars, the bars’ length represent the 95% confidence interval; **F** 16 DEGs were selected through lasso Cox regression by tenfold cross validation, red represent the risk DEGs and blue represent the protective DEGs; **G** 10 DEGs were finally gathered through step-wise multivariate Cox regression to construct the risk signature

Firstly, a total of 398 DEGs were identified between the paracancerous and tumor samples. The heatmap and volcano plot were showed in Fig. 2A, B. Functional enrichment analysis indicated that the DEGs are mainly associated with mRNA processing and RNA splicing biological process, in addition, KEGG results presented these DEGs mainly participate in mRNA surveillance and spliceosome pathway (Fig. 2C, D). Secondly, we performed a series of bioinformatics methods to select candidate genes and establish the risk signature. TCGA-CESC cohort was randomly separated into a training cohort (208 patients) and a testing cohort (88 patients), the detailed information was listed in Additional file 1: Table S3. Univariate Cox regression showed that 26 RBPs were significantly associated with prognosis among CESC (Fig. 2E, Additional file 1: Table S4). Lasso regression indicated that the partial likelihood deviance was minimal when log(lambda) equal to − 3.846, and 16 genes were obtained for further analysis (Fig. 2F, Additional file 1: Figure S1). The 10-RBPs signature was finally constructed by multivariate stepwise Cox regression (Fig. 2G). The detailed information of Cox result was presented in Table 1 and the risk score was calculated as mentioned above.

**Table 1:** Multivariate Cox results of the 10 RBPs in the risk signature

Gene	Coefficient	Hazard ratio	Lower 95%CI	Upper 95%CI	*P* value
AIMP2	0.431	1.539	1.080	2.193	0.017
ANGEL2	0.490	1.632	1.159	2.300	0.005
DDX26B	− 0.683	0.505	0.340	0.750	< 0.001
GAPDH	0.285	1.330	0.998	1.774	0.052
HENMT1	− 0.374	0.688	0.510	0.927	0.014
PRPF40B	0.544	1.723	1.188	2.501	0.004
RBM38	− 0.437	0.646	0.448	0.931	0.019
RNASEH2A	− 0.359	0.698	0.503	0.969	0.032
SNRPN	− 0.459	0.632	0.475	0.841	0.002
LRRFIP1	0.265	1.304	0.946	1.797	0.105

*CI* confidence interval

### 10-RBPs signature performed satisfactory prognostic ability both in training cohort and testing cohort

**Fig. 3:**
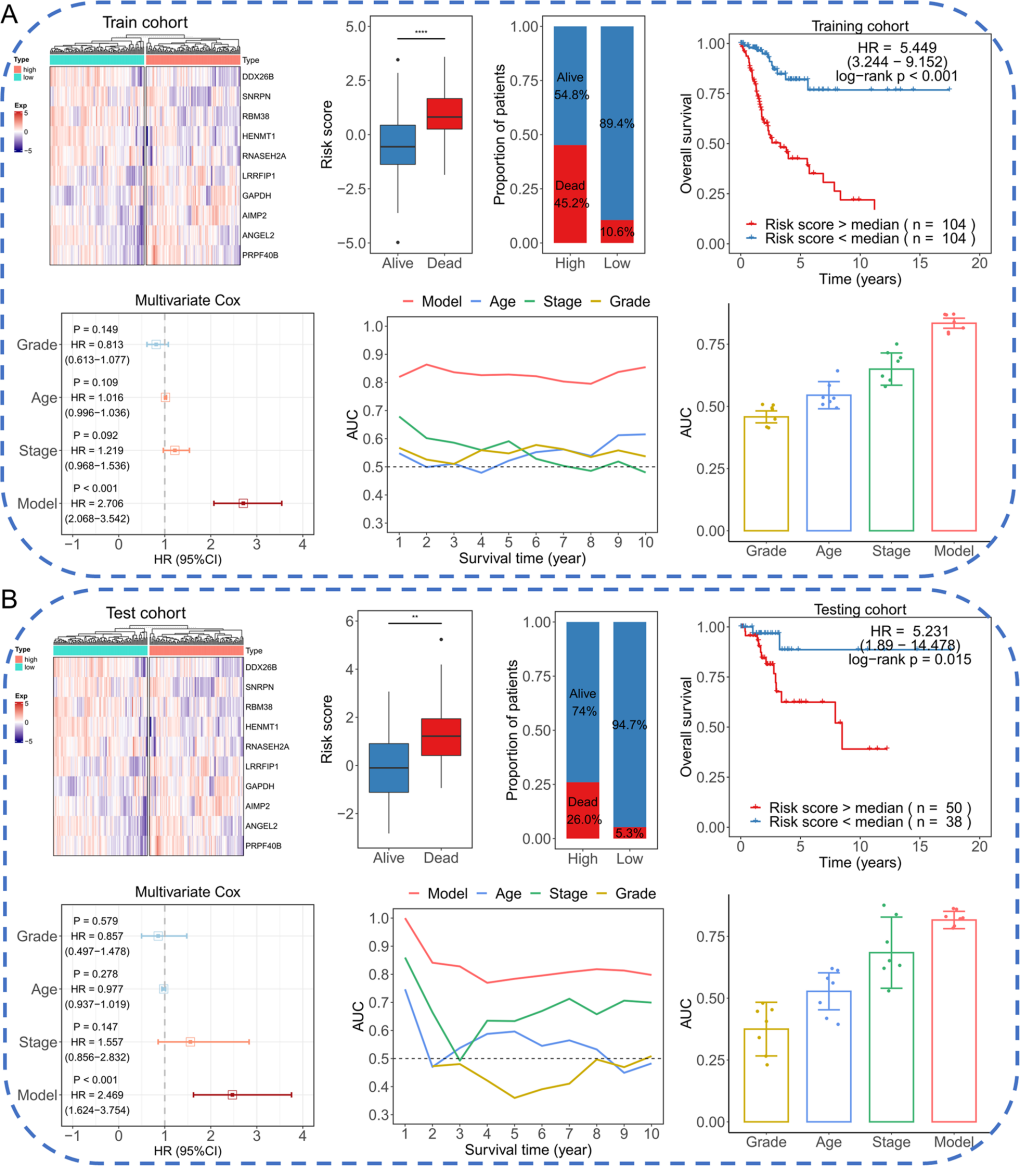
Evaluation the 10-RBPs signature in training cohort (**A**) and testing cohort (**B**). The transcript difference of 10 RBPs between high-risk group and low-risk group were presented in heatmap; Boxplot to show the difference of risk value between alive patients and dead patients; Stacked column chart was employed to present the proportion of alive and dead patients between high-risk group and low-risk group; Survival plot was conducted to exhibit the prognostic difference between high-risk group and low-risk group; Forest plot presented the multivariate Cox regression of 10-RBPs signature and clinical parameters; Prediction ability of 10-RBPs signature and other clinical factors were displayed in tAUC curves; Barplot to show the difference of AUC towards various indicators

Next, we evaluated the 10-RBPs signature in the training cohort firstly. As shown in Fig. 3A, the transcript levels of these 10 RBPs were presented in a heatmap, the five protective genes were higher expressed in low-risk group, and the remaining five risk genes were higher expressed in high-risk group. The risk score was significantly higher in dead patients compared with alive patients (*P* < 0.001). Meanwhile, the proportion of dead patients in high-risk group was much higher. Survival plot indicated that the patients in high-risk group exhibit significantly shorter overall survival time (*P* < 0.001). Multivariate Cox regression suggested that the 10-RBPs signature served as an independent prognostic factor among CESC patients (*P* < 0.001). tAUC plot showed the 10-RBPs signature was much more accurate than other clinical parameters including age, stage and grade. The bar plot was used to show the quantitative results of tAUC. Furthermore, the 10-RBPs signature was validated in testing cohort and similarity results were presented in Fig. 3B.

### The nomogram could effectively predict the prognosis among TCGA-CESC patients

**Fig. 4:**
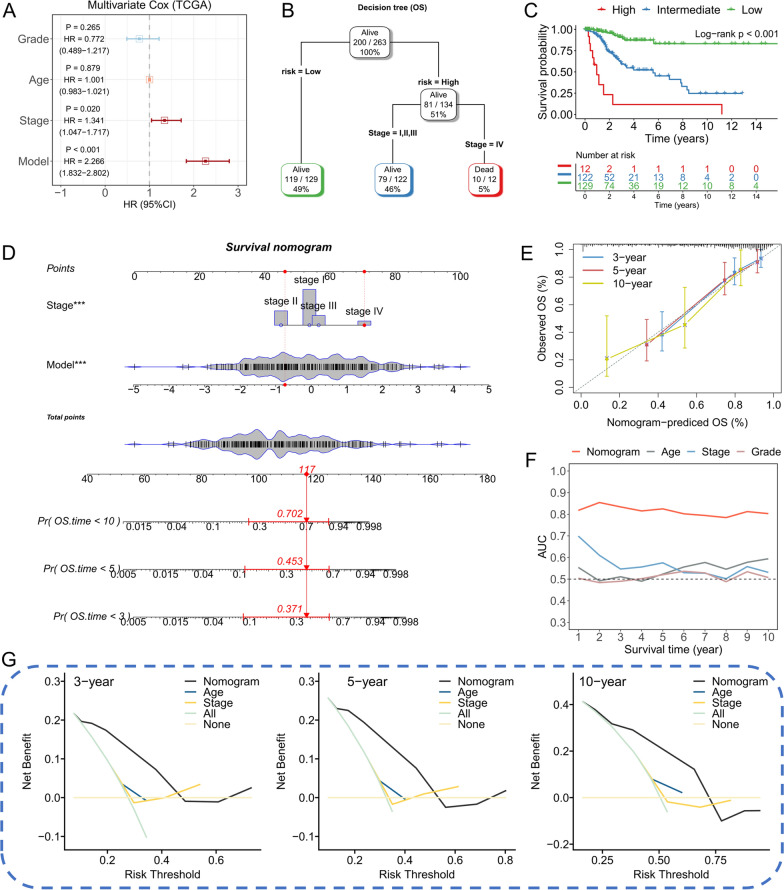
Assessment the clinical application of the 10-RBPs signature in TCGA-CESC cohort. **A** Forest plot to show the multivariate Cox regression of age, grade, stage and risk signature; **B** A decision tree was established by combining the risk signature and pathological stage for the purpose of optime the risk stratification; **C** Significant survival difference was observed between the three subgroups; **D** Details of the nomogram, red arrow represent the calculate process of a random selected patient; **E** Calibration curves of the nomogram; **F** Comparison of the tAUC between nomogram, age, grade and stage; **G** DCA curves to show the net-benefit of nomogram at 3-year, 5-year and 10-year time point

To explore the clinical application of the 10-RBPs signature, we combined the training cohort and testing cohort and 263 CESC patients with full-scale clinical information were extracted. Multivariate Cox regression indicated the pathological stage (*P* = 0.020) and risk score (*P* < 0.001) were both remarkably associated with prognosis among TCGA-CESC patients (Fig. 4A). Hence, the stage and risk scores were submitted to establish the decision tree. As shown in Fig. 4B, three different subgroups were identified based on two major components in which the risk score showed the most powerful effect. Patients with lower risk scores were labeled as “low-risk”, while higher risk scores & stage I–III and higher risk & stage IV were labeled as “intermediate-risk” and “high-risk”, respectively. Survival analysis presented significant differences between the subgroups (Fig. 4C). To quantify the individual risk assessment with CESC, a nomogram was developed to achieve this goal (Fig. 4D). The red arrow showed an illustration of the calculation process. Calibration curves indicated that the nomogram performed good performance in predicting prognosis (Fig. 4E). tAUC illustrates the nomogram acts with much higher accuracy in prognosis prediction than else clinicopathological features (Fig. 4F). DCA curves indicated the nomogram brings about more net benefit for CESC patients than other clinical parameters in different cut-offs of survival time (Fig. 4G).

### 10-RBPs signature may participate in various cancer-related pathways and play a vital role in tumor progression

**Fig. 5:**
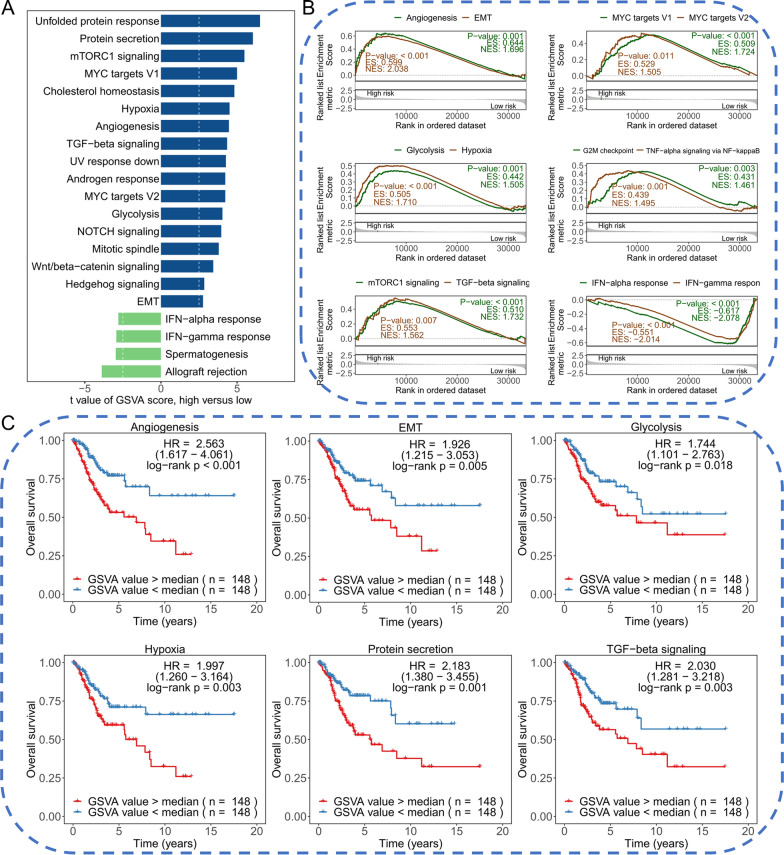
Functional enrichment analysis between high-risk group and low-risk group. **A** Hallmark gene sets were quantified by “GSVA” and compared between high-risk group and low-risk group; **B** GSEA to explore the difference of pathway between high-risk group and low-risk group; **C** Cancer-related pathways presented significant survival difference in CESC

To comprehensively explore the biological functions of the risk signature, we performed enrichment analysis in TCGA-CESC based on Hallmark gene sets. Firstly, the 50 Hallmark gene sets were quantified by “ssgsea” and the results showed most gene sets were significantly higher in high-risk group compared with low-risk group, including hypoxia, glycolysis and EMT, et al. (Fig. 5A). GSEA results indicated most cancer-related pathways were enriched in high-risk group, including angiogenesis, EMT, hypoxia, et al., and the immune-related pathways were enriched in low-risk group, including IFN-alpha response and IFN-gamma response (Fig. 5B). Survival analysis presented that these cancer-related pathways were positively associated with poor prognosis, which is consistent with the above results (Fig. 5C). In addition, the landscape of somatic mutation was also explored between different risk groups. The top 20 mutated genes in different groups were presented and compared (Additional file 1: Figure S2A, B). PIK3CA showed the highest mutation frequency in high-risk group, while TTN showed the highest mutation frequency in low-risk group. Fisher’s exact test revealed that PDE3A, STK11, APC, BAHCC1, DSCAML1, TCOF1, SPEN and DNAH3 were higher mutated in the high-risk group compared with low-risk group (*P* < 0.05, Additional file 1: Figure S2C). The lollipop plot showed the different mutational sites of PDE3A (Additional file 1: Figure S2D). Besides, co-occurrence and mutually exclusive mutations were also investigated. From the results, we know that most co-occurrence patterns happened in high-risk group, which indicated a potential common effect induced by their mutations (Additional file 1: Figure S2E, F).

### 10-RBPs signature showed a significant relationship with immune cell infiltration and immunotherapy response

**Fig. 6:**
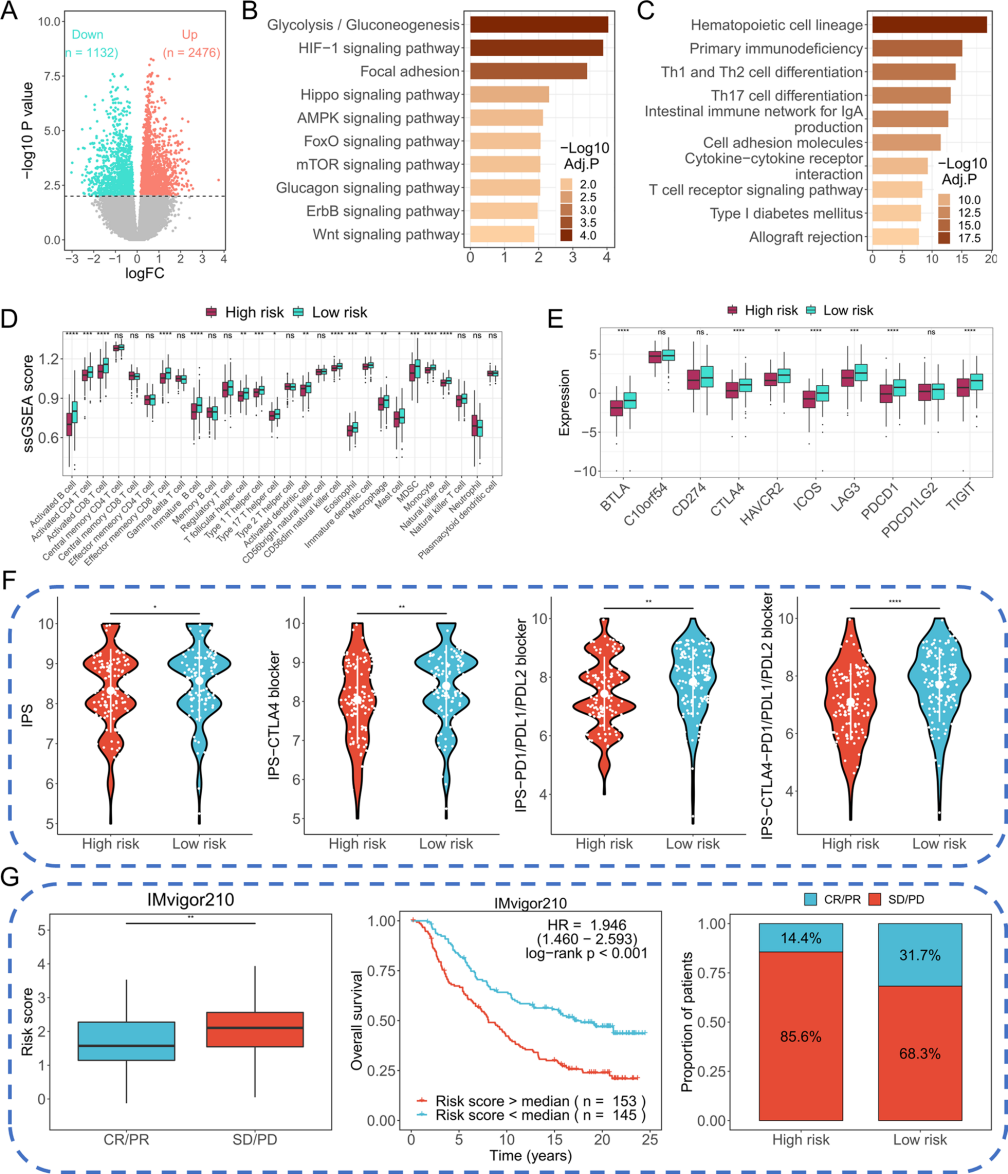
The effect of 10-RBPs on immunotherapy. **A** A total of 3608 DEGs were showed in volcano plot (*P* < 0.05); **B** KEGG enrichment of the up-regulated DEGs; **C** KEGG enrichment of the down-regulated DEGs; **D** Comparison of the 28 immune cells between high-risk group and low-risk group; **E** Comparison the transcript difference of 10 immune checkpoints between high-risk group and low-risk group; **F** Violin plot presented the differences of IPS scores between high-risk group and low-risk group; **G** Exploration of the immunotherapy response of 10-RBPs signature in IMvigor210 cohort

A total of 3608 DEGs were identified between high-risk group and low-risk group, the results were presented in volcano plot (Fig. 6A). Enrichment analysis indicated the up-regulated genes were mainly participate in cancer-related pathways, including glycolysis/gluconeogenesis, HIF-1 signaling pathway, focal adhesion and Hippo signaling pathway, et al. (Fig. 6B). The down-regulated genes were enriched in immune-related pathways, for example, the T cell receptor signaling pathway (Fig. 6C). These results indicated the 10-RBPs signature may influence the tumor-immune interaction. Hence, we quantified and compared the infiltration of 28 immune cells between different risk groups. As shown in Fig. 6D, most immune cells presented higher infiltration in low-risk group. Similarly, the immune checkpoint genes were significantly higher expressed in low-risk group (Fig. 6E). Furthermore, the IPS, IPS-CTLA4 blocker, IPS-PD1/PD-L1/PD-L2 blocker and IPS-CTLA4- PD1/PD-L1/PD-L2 blocker were remarkably elevated in low-risk group, indicated the patients in low-risk group may present more sensitivity to immunotherapy response (Fig. 6F). Finally, the IMvigor210 cohort was used to evaluate the prognostic ability of 10-RBPs signature. As shown in Fig. 6G, boxplot showed the risk score was significantly higher in “SD/PD” group compared with “CR/PR” group, from which we know the risk score was negatively associated with immunotherapy response. Survival plot declared the 10-RBPs signature also act good performance in IMvigor210 cohort (*P* < 0.001). As expected, the high-risk group possesses a higher proportion of patients who showed “SD/PD” response to immunotherapy.

### 10-RBPs signature served as a potential biomarker in chemotherapy resistance

**Fig. 7:**
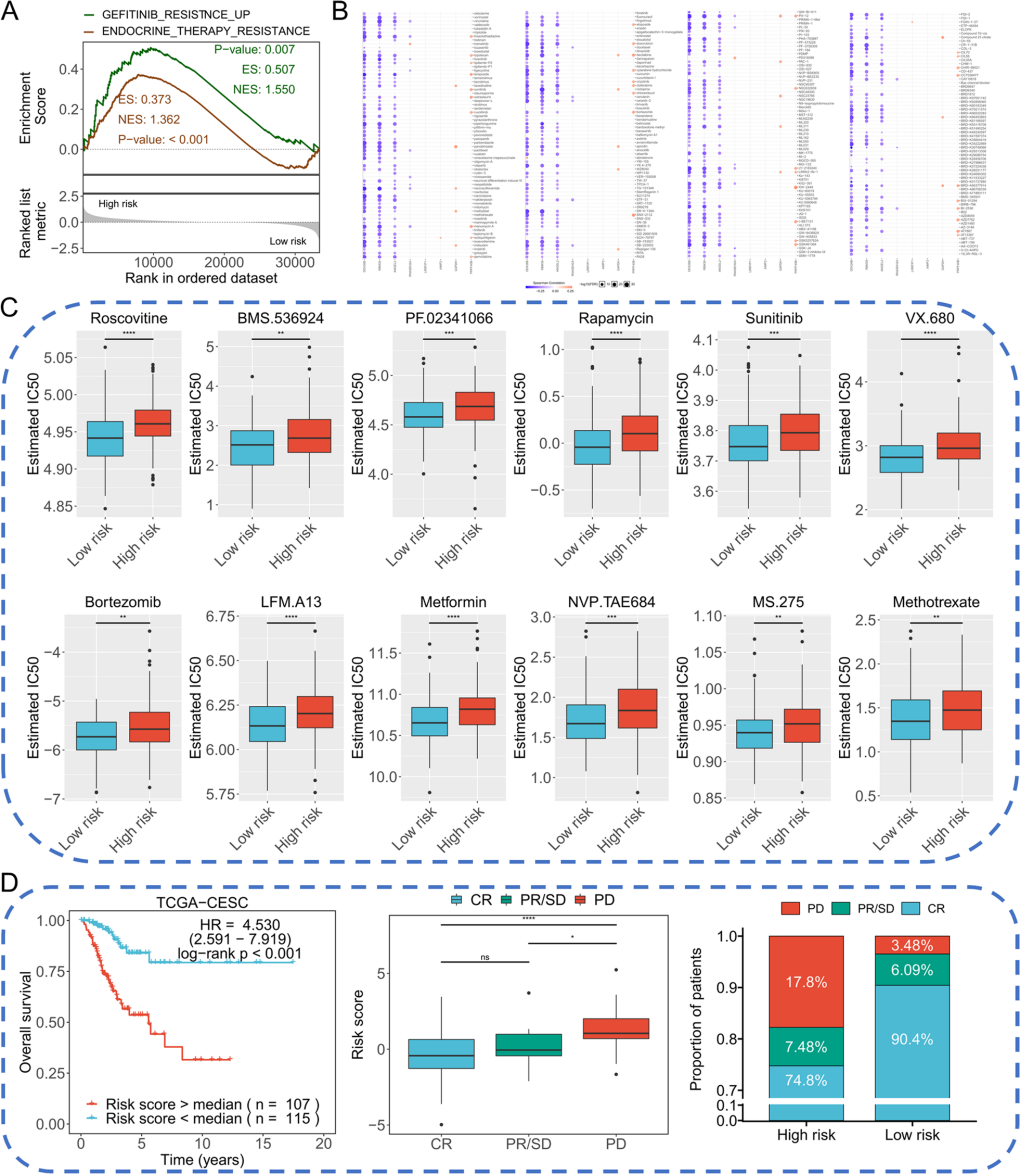
The effect of 10-RBPs on chemotherapy. **A** GSEA showed the high-risk group was positively associated with chemotherapy resistance pathways; **B** Correlation between transcript expression and IC50 data retrieved from GDSCLike database; **C** Comparison the estimated IC50 data between high-risk group and low-risk group in TCGA-CESC; **D** Evaluate the chemotherapy response of 10-RBPs in CESC

GSEA results indicated that two chemotherapy-related pathways were enriched in high-risk group, including gefitinib resistance pathway and endocrine therapy resistance pathway (Fig. 7A). GSCALike web tool provided the Spearman correlation between gene expression and IC50 drug data, the positive correlation means the gene’s high expression is resistant to the drug, and vice versa. The results presented that DDX26B, RBM38 and ANGEL2 were negatively correlated with IC50 data, while PRPF40B was positively correlated with IC50 data, providing certain evidence for the effect of 10-RBPs on chemotherapy resistance (Fig. 7B). In addition, we performed chemotherapy prediction in TCGA-CESC and the results indicated the high-risk patients showed higher IC50 in several chemotherapy drugs, including Roscovitine, BMS.536924, PF.02341066, Rapamycin, Sunitinib, VX.680, Bortezomib, LFM.A13, Metformin, NVP.TAE684, MS.275 and Methotrexate (Fig. 7C). Besides, a total of 222 TCGA-CESC patients who received adjuvant therapy were selected and significant survival difference was observed between different risk groups among these patients. We further divided these 222 patients into three subgroups labeled “CR”, “PR/SD” and “PD”, respectively. Results indicated the “PD” subgroup possessed highest risk score compared with other subgroups, while the “CR” subgroup presented the lowest risk score. Meanwhile, the proportions of “PD” and “CR” patients in high-risk group and low-risk group were significantly different (Fig. 7D).

### PRPF40B was up-regulated in CESC tissues and cell lines

**Fig. 8:**
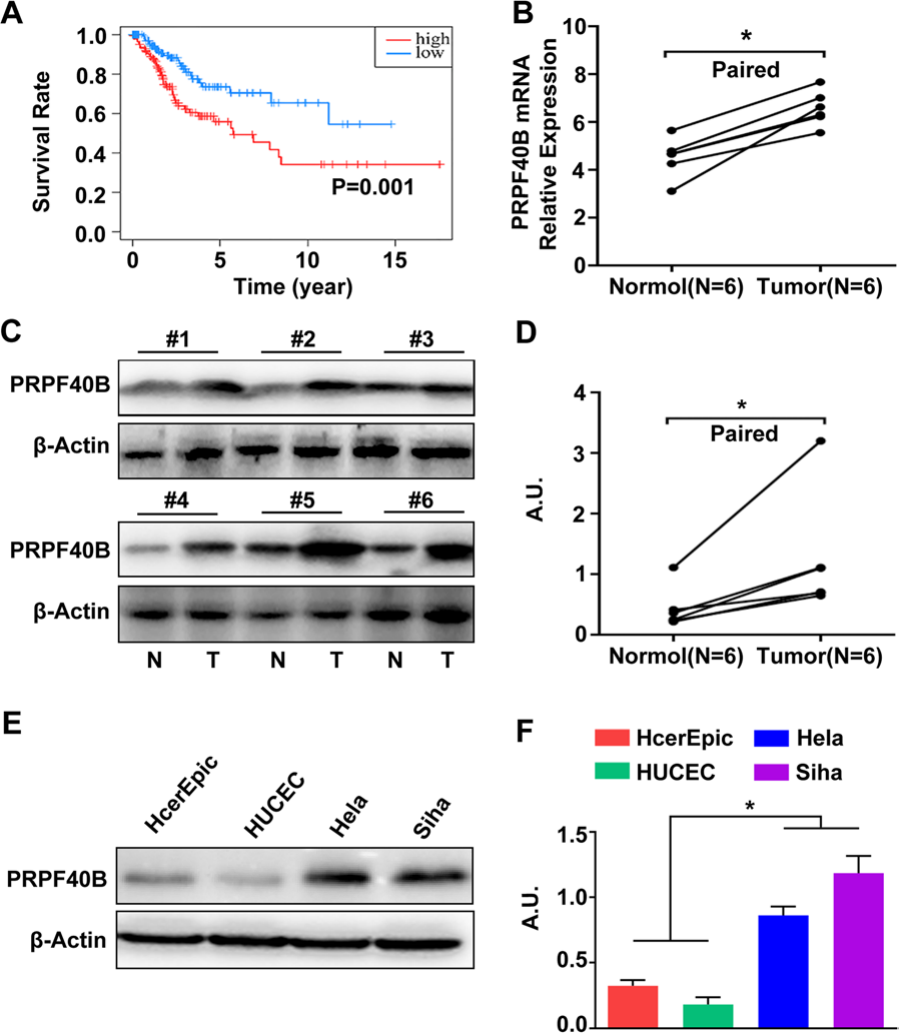
Verify the expression level of PRPF40B in CESC tissues and cell lines. **A** Survival plot of PRPF40B in TCGA database; **B** the transcript level of PRPF40B in CESC tissues and paired normal tissues; **C**, **D** the translation level of PRPF40B in CESC tissues and paired normal tissues, and the quantitative results; **E**, **F** the translation level of PRPF40B in four cervical cell lines and the quantitative results. Values are expressed as the mean ± SD (n = 3); *P < 0.05 versus vehicle-treated control group; A.U. arbitrary units

From the above results, we observed PRPF40B may act as a prognostic biomarker in CESC. Hence, the study verified the expression level of PRPF40B in clinical tissues and CESC cell lines. Firstly, survival analysis indicated that higher expression of PRPF40B showed a positive association with worse OS in TCGA database (Fig. 8A). Secondly, results of RT-qPCR indicated that the relative mRNA expression of PRPF40B was significantly higher in CESC tissues compared with paracancerous tissues (*P* < 0.05, Fig. 8B). The translation level of PRPF40B was verified through western blot (*P* < 0.05, Fig. 8C, D). In addition, the protein level of PRPF40B was also increased in CESC cell lines Hela and Siha (*P* < 0.05, Fig. 8E, F).

### PRPF40B accelerates the proliferation, migration and invasion of CESC in vitro

**Fig. 9:**
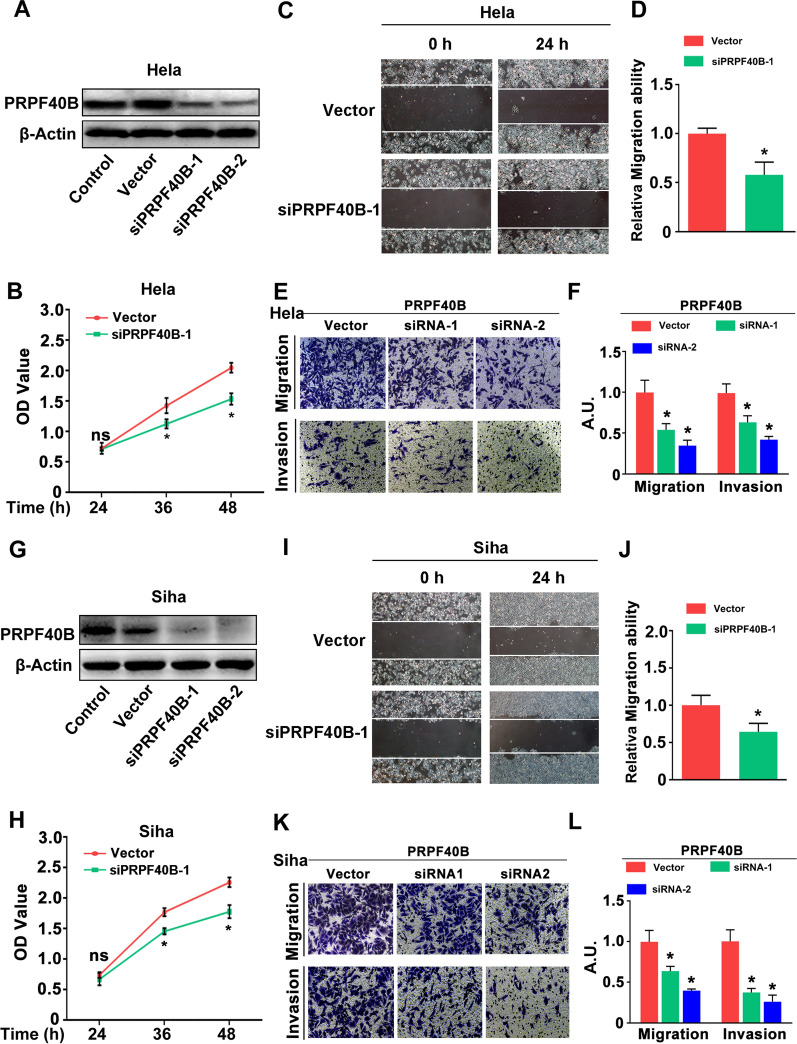
Investigate the biological function of PRPF40B in CESC cell lines. **A**, **G** Western-blot displayed the siPRPF40B model in Hela and Siha cell lines; Proliferation assay of vector and siPRPF40B in Hela cell (**B**) and Siha cell (**H**); Wound-healing assay of vector and siPRPF40B in Hela cell (**C**, **D**) and Siha cell (**I**, **J**); Transwell assay of vector and siPRPF40B in Hela cell (**E**, **F**) and Siha cell (**K**, **L**). Values are expressed as the mean ± SD (n = 5); *P < 0.05 versus vehicle-treated control group

To further evaluate the biological functions of PRPF40B, we conduct experimental validation for this hypothesis. Western-blot presented that the PRPF40B was successfully knockdown in Hela and Siha cell lines (Fig. 9A, G). CCK-8 assay suggested that the proliferation of Hela and Siha cells was significantly suppressed in the siPRPF40B group (*P* < 0.05, Fig. 9B, H). The wound healing assay indicated that siPRPF40B significantly inhibits the growth rate of Hela and Siha cells (Fig. 9C, D, I, J). Consistent with the above results, transwell assay suggested the migration and invasion ability of these two cells were obviously suppressed after siPRPF40B was treated (Fig. 9E, F, K, L). These results declared that PRPF40B acts as an oncogene in CESC, which is in accordance with the prognostic effect, even though the underlying mechanism is unknown.

## Discussion

Emerging evidence suggested that RBPs played vital roles during tumorigenesis and progression, however, little is known about the transcript pattern and effect of RBPs in CESC. Hence, we developed a robust 10-RBPs signature and investigated the potential functions of hub genes retrieved from the risk model. The 10-RBPs signature could serve as a novel biomarker for CESC patients toward prognosis, immunotherapy and chemotherapy. Besides, the nomogram could transform the categorical variables into quantitative values and provide precise risk assessment among CESC patients. Notably, in *vitro* experiments indicated that the PRPF40B acts as a risk biomarker in CESC cell lines. All these discoveries demonstrated the 10-RBPs have satisfactory predictive ability on the OS of CESC patients.

Our study found 398 RBPs showed differential expression between paracancerous tissues and CESC tissues, functional enrichment suggested these RBPs mainly participate in mRNA processing, RNA splicing and regulation of translation. Previous studies have found that abnormal regulation of translation, RNA processing and RNA metabolism are the driving force for the occurrence and development of human diseases [16, 17], which is consistent with our conclusions. Previous studies pointed out that the prognosis model of CESC patients based on histone family, miRNA and lncRNA performed well in predicting the survival rate of CESC [18–20], however, models based on the role of RBPs in CESC and its prognostic effects have not been explored and constructed. Considering the limited ability of a single RBP to predict prognosis, this study built a prediction model based on 10 RBPs (DDX26B, SNRPN, RBM38, HENMT1, RNASEH2A, LRRFIP1, GAPDH, AIMP2, ANGEL2 and PRPF40B) through a series of bioinformatics analysis. Our results showed that CESC patients with high expression of AIMP2, ANGEL2 and PRPF40B had poor OS, while those with high expression of DDX26B, SNRPN, HENMTI, RBM38 and RNASEH2A had better OS. RBM38 can change the stability and translation of targeted mRNA, thus affecting cell proliferation, cell cycle arrest, myogenic differentiation and other biological processes, and is a potential biomarker and therapeutic target for human tumors [21]. In addition, AIMP2 is a non-enzymatic component required by multi-tRNA synthetase complex. Its alternative splicing variant AIMP2-DX2 can damage the activity of AIMP2 and is related to carcinogenesis, and the proportion of AIMP2-DX2/AIMP2 is closely related to the main cancer signal pathway and poor prognosis [22]. The human PRPF40B gene can regulate hundreds of alternative splicing sites and inhibit hypoxia expression signals, which plays an important role in the development of human tumors [23]. The above evidence showed that the functions of RBPs were closely related to human tumors, and can be used to build models and support our research results.

RBPs signatures have been developed in several human cancers, including hepatocellular carcinoma, endometrial cancer, head and neck squamous cell carcinoma, lung adenocarcinoma, et al. [24–27]. This study established the 10-RBPs signature in CESC, furthermore, a nomogram was constructed based on the risk signature and pathological stage. Compared with age, pathological stage and grade, the tAUC of the nomogram was significantly higher and the mean value reached upon 0.8. Besides, the decision tree indicated the 10-RBPs signature was the dominant influencing factor for CESC’s prognosis. GSEA presented most classic cancer-related pathways were obviously enriched in high-risk group compared with the low-risk group, including angiogenesis, EMT, glycolysis, hypoxia, cell cycle, mTORC1 and TGF-beta signaling pathway, which proved the 10-RBPs signature had the potential to be a reliable biomarker for CESC from the side. Enhanced glycolysis was the additional energy source for tumor proliferation and progression, for example, IGF2BP2 performed as a RBP and could regulate the m6A manner of MYC and thus promoted the aerobic glycolysis, migration and proliferation in CESC [28]. mTORC1 could regulate the invasion, proliferation and EMT of CESC cells through PI3K/AKT/mTORC1 pathway as one of the signaling complexes of mTOR [29]. Wang’s study identified 19 invasion-related genes and clustered the CESC into two molecular subtypes, and found TGF-beta signaling pathway was positively associated with the poor prognostic subtype [30]. In addition, our study performed survival analysis of these cancer-related pathways in CESC. The results indicated most cancer-related pathways were significantly related to worse prognosis, which was consistent with the above results and other research, suggesting the tremendous exploration value and potential mechanism of 10-RBPs signature in CESC.

Remarkably, our signature may serve as a potential biomarker for immunotherapy and chemotherapy response. Functional enrichment analysis indicated that a variety of cancer-related pathways and chemotherapy-resistance pathways were enriched in high-risk group, while immune-related pathways were enriched in low-risk group, which is consistent with the previous results of our study. Previous reports demonstrated that immune escape is essential for tumor survival and progression, and may induce immunotherapy resistance [31]. Here, we found the relative infiltration of immune cells and the transcript level of immune checkpoints were both significantly increased in low-risk group. Meanwhile, the IPS scores were remarkably elevated in low-risk group, suggesting the patients in low-risk group might present more sensitivity to immunotherapy response.

It is known that post-transcriptional regulation is a dynamic and continuous process, but it is not clear whether the changes of RBPs are sufficient to reflect the functions. Therefore, our work had some limitations. Firstly, the prognosis model was only based on TCGA cohort data, and needs to be verified in clinical patient cohorts and multi-center prospective study. Secondly, further experimental studies in vitro and in vivo are needed to clarify the molecular mechanism to better carry out clinical practice.

In conclusion, this study systematically explored the functions and potential prognostic value of RBPs in CESC, established a risk signature based on 10 RBPs and constructed a nomogram, aiming to provide new reference information for the individualized treatment and clinical outcome prediction of CESC patients. The 10-RBPs signature may act as a novel indicator for immunotherapy and chemotherapy, which could fill the gaps in CESC’s treatment strategy and supply promising research topics for future research.

## Materials and methods

### Data acquisition and procession

The level 3 of TPM normalized RNA sequencing data of CESC and paracancerous cervical tissues were obtained from The Cancer Genome Atlas (TCGA) and Genotype-Tissue Expression (GTEx) database through UCSC online webtool (https://xenabrowser.net/datapages/), respectively. A total of 10 paracancerous cervical samples and 296 CESC samples were acquired after removing the samples with missing survival times. Besides, 1542 RBPs were gathered from Gerstberger’s review [10] and used for further bioinformatics analysis. In addition, the transcriptome profile of 298 metastatic urothelial cancer patients treated with anti–PD-L1 agent (atezolizumab) and corresponding clinical outcomes was collected from “IMvigor210CoreBiologies” package [32, 33], and used to verify the immunotherapy response of our signature.

### Model construction and validation

Differentially expressed RBPs were identified through “limma” package with the threshold as: |log2FC|> 1 & adjust *P* value < 0.05. Then, the TCGA-CESC cohort was divided into a training cohort and a testing cohort by “caret” package with a relative proportion of 7:3. In the training cohort, candidate genes were selected through a sequential procedure of univariate Cox regression, Lasso regression and multivariate stepwise Cox regression to establish the risk signature. The risk score was calculated as the sum of the product of gene expression and regression coefficient. Then, the risk signature was validated in the training cohort and testing cohort, respectively.

### Functional enrichment analysis

Biological process and Kyoto Encyclopedia of Genes and Genomes (KEGG) pathway enrichment analysis were performed by “clusterProfiler” package [34] with the adjust *P* value < 0.05 as the selection criteria. Gene Set Enrichment Analysis (GSEA) was employed to explore the diverse potential pathways between different risk groups, and the Hallmark gene sets and chemical and genetic perturbations gene sets were chosen as the reference gene sets, respectively [35–37].

### Single nucleotide variation analysis

The masked somatic mutation profile of CESC was downloaded from TCGA database through “TCGAbiolinks” package and the landscape of top 20 mutated genes was presented and compared between different risk groups by “maftools” package [38]. The mutation sites of specific gene were shown by a lollipop plot and somatic interactions between different groups were performed by pair-wise Fisher’s exact test.

### Immunotherapy response analysis

Firstly, the DEGs between the high-risk group and the low-risk group were explored by the “limma” package with the selection criterion as adjust *P* < 0.01 and used to display KEGG pathways through the “clusterProfiler” package. Secondly, the list of gene markers of 28 immune cells was obtained from Bindea’s study [39] and employed to quantify the relative infiltration of 28 immune cells by “GSVA” package through “ssgsea” method. Besides, the cellular characteristics of immune infiltration indicate that tumor genotype determines the immune phenotype and tumor escape mechanism. Charoentong [40] has developed a quantitative scoring scheme called Immunophenotypic Score (IPS), which is a better predictor of antibody responses to cytotoxic T lymphocyte antigen 4 (CTLA-4) and anti-programmed cell death protein 1 (anti-PD-1). We further acquired the IPS of CESC samples from The Cancer Immunome Atlas (TCIA) (https://tcia.at/home).

### Chemotherapy response analysis

The relationship between IC50 data of multiple molecules and the gene signature in CESC was conducted through GSCALite [41] webtool (http://bioinfo.life.hust.edu.cn/web/GSCALite/). Meanwhile, we also investigated the IC50 data of variety chemotherapy drugs in different risk groups through “pRRophetic” package, which construct the ridge regression model according to GDSC (www.cancerxgene.org/) cell line expression profile and TCGA gene expression profile to predict drug IC50 [42].

### Clinical sample collection

Six paired clinical specimens of CESC and paracancerous cervical tissues were collected from patients who were surgically removed in the Obstetrics and Gynecology Department of the Southwest Hospital in December 2022 to explore the different expression of hub RBPs. This study has obtained the written informed consent of all patients. The collection of clinical specimens for research has been approved by the Ethics Committee of the Southwest Hospital [KY2022151] in accordance with the ethical standards as laid down in the 1964 Helsinki Declaration and its later amendments or comparable ethical standards. The detailed methods were as follows: the collection of specimens during the surgery was taken within 30 min and three specimens were collected for each patient. The paracancerous tissues were more than 3 cm far away from the edge of the tumor and each specimen was about 0.5 cm × 0.5 cm. The specimens were then directly placed in a frozen storage tube, marked and soaked in liquid nitrogen, and then stored in a refrigerator at − 80 °C.

### Cell culture and cell transfect

Human cervical epithelial cell lines HcerEpic and HUCEC, and human CESC cell lines Hela and Siha were purchased from the cell bank of the Committee for the Preservation of Typical Cultures, Chinese Academy of Sciences. Cells were cultured in 37 °C, 5%CO_2_ incubator and RPMI-1640 medium containing 10% fetal bovine serum was added to re-suspend the cells after the cells were recovered. Then trypsin digestion and passage were carried out when the cells fused to about 80%. Cells in the logarithmic growth phase were selected and re-seeded into 6 well plates (3 × 10^5^/well). When the cell density reached 50%–70%, the transfection was carried out according to the instructions of the Lipofectamine 3000 transfection kit. The sequences of siRNAs of PRPF40B were presented in Additional file 1: Table S1.

### Real-time quantitative polymerase chain reaction (RT-qPCR)

Total RNA from CESC tissues was extracted using TRIZOL reagent and quantified using ultraviolet spectrophotometry. The cDNA was synthesized using the TAKARA reverse transcription kit. The RT-qPCR was conducted through SYBR-Green detection kit. The reaction conditions were as follows: 94 °C for 4 min, then 40 cycles were conducted at 94 °C for 30 s, 58 °C for 30 s, and 72 °C for 30 s. β-Actin was chosen as the internal reference and the relative expression was calculated using 2^−ΔΔt^ method. The primer sequences were presented in Additional file 1: Table S2. Each experiment was conducted triplicate and mean ± standard deviation (SD) was employed to represent the quantitative value.

### Western blot

The collected clinical specimens and CESC cell lines were subjected to ice bath lysis with human RIPA lysis buffer for 30 min, centrifugation at 12,000 r/min for 20 min, and the supernatant was collected. The protein samples in the supernatant were denatured, separated by 10% sodium dodecyl sulfate polyacrylamide gel electrophoresis, and transferred to the polyvinylidene fluoride film. After sealing with 5% skim milk powder for 2 h, the PRPF40B antibody was added to incubate overnight. Then rinse the membrane with TBST for 3–5 times, and incubate it with horseradish peroxidase (HRP-) labeled goat anti rabbit IgG antibody (1:10,000) for 1 h. β-Actin (1:5000) was selected as an internal reference. Then use ECL luminescence kit to detect protein bands on the membrane, and plot and analyze the relative expression of each protein in ImageJ software v1.53c (NIH, Bethesda, MD, United States).

### Proliferation assay

The transfected Hela and Siha cells were added to 96 well plates (1 × 10^4^/mL, 200 μL) and cultured at 37 °C for 24, 36 and 48 h. Then, 10 μL CCK-8 solution is added to each hole. After incubation at 37 °C for 2 h, the microplate reader was used to detect the absorbance (OD) value at 450 nm wavelength. Each experiment was repeated at least five times and the OD value was represented as mean ± SD.

### Wound healing assay

The Hela and Siha cells in each group were inoculated into 6 well plates and cultured overnight in a humid environment at 37 °C. When cells reached 100% confluence, use the 10 μL suction nozzle of the pipette slightly scratches the monolayer cells linearly, and the cells are washed with phosphate buffered saline (PBS) for three times. Cells were incubated at 37 °C for 24 h in RPMI-1640 medium without FBS, and cell migration was observed and photographed at 0 h and 24 h with phase contrast microscope. Scratch healing rate was calculated as: (0 h scratch width—24 h scratch width)/0 h scratch width × 100%.

### Transwell assay

Transwell cells coated with or without matrix glue mixture were used to evaluate cell invasion and migration. The stably transfected cells were seeded (3 × 10^4^/well) in the upper chamber of serum free medium, and the lower chamber used the medium containing 10% FBS as the chemotactic agent. After incubation for 24 h, the cells at the bottom of the chamber were fixed with 4% paraformaldehyde for 15 min, stained with 0.1% crystal violet for 15 min, and photographed under the microscope. Five areas were randomly selected from each hole to count the invasive or migratory cells.

### Other bioinformatics and statistical analysis

GraphPad Prism 8.0 (GraphPad Software Inc, San Diego, CA) and R software (version 4.2.1, http://r-project.org) were used to analyze data and plot graphs. Survival differences between different risk groups were compared through “survival” package and the median risk score was used as cut-off value. Time-dependent area under the curve (tAUC) was performed through “riskRegression” package to distinguish the prognostic efficiency between risk signature with other clinical parameters. Recursive regression analysis was employed to construct the decision tree by “rpart” package. The nomogram was established to quantify the risk value of patient by combining pathological stage and risk signature through “nomogramEx” package. Calibration curves were conducted to examine the consistency between predictive prognosis and ideal prognosis. Decision curve analysis (DCA) was applied to explore the net profit between variety factors by “ggDCA” package. Quantitative variables were compared by *t* test or *Wilcoxon* test and survival plots were compared by Log-rank test. Unless otherwise specified, *P* < 0.05 was considered as statistically significant.

## Novelty & impact statements

This research constructed and validated a robust RNA-binding proteins related signature upon cervical cancer patients’ outcome, which also exhibit satisfactory prediction ability in immunotherapy and chemotherapy response. In addition, PRPF40B was identified as hub gene based on our results through the signature. Down-regulated PRPF40B could inhibit the proliferation, invasion and migration of cervical cancer cell lines in vitro experiments. Our research provided novel insight in cancer biomarker selection.

### Supplementary Information

**Additional file 1: Table S1.** siRNA sequence of PRPF40B. **Table S2.** Primer sequence of RT-qPCR. **Table S3.** Detailed information of training and testing cohort. **Table S4.** Univariate Cox result in the training cohort. **Figure S1.** The process of lasso cox regression. (**A**) The partial likelihood deviance was minimal when log(lambda) equal to -3.846, and 16 DEGs were obtained for further analysis; (**B**) 16 DEGs presented non-zero coefficient when log(lambda) equal to -3.846. **Figure S2.** Mutational analysis of CESC between different risk groups. (**A**) Top 20 mutated genes in high-risk group; (**B**) Top 20 mutated genes in low-risk group; (**C**) Significant mutated genes between high-risk group and low-risk group; (**D**) Lollipop chart to show the different mutate sites of PDE3A; (**E**) Co-occurrence and mutually exclusive patterns in high-risk group; (**F**) Co-occurrence and mutually exclusive patterns in low-risk group.

## Data Availability

The transcript profile, clinical information and single nucleotide variation data of CESC were downloaded from UCSC database (https://xenabrowser.net/datapages/). The raw data and script of R code was uploaded as additional materials.
