# Supplementary material for: RNA-binding proteins signature is a favorable biomarker of prognosis, immunotherapy and chemotherapy response for cervical cancer
Source: Cancer Cell Int. 2024 Feb 21;24:80. doi: 10.1186/s12935-024-03257-w (PMC10882920; doi:10.1186/s12935-024-03257-w)
Supplement: Supplementary file 1 — Additional file 1: Table S1. siRNA sequence of PRPF40B. Table S2. Primer sequence of RT-qPCR. Table S3. Detailed information of training and testing cohort. Table S4. Univariate Cox result in the training cohort. Figure S1. The process of lasso cox regression. (A) The partial likelihood deviance was minimal when log(lambda) equal to -3.846, and 16 DEGs were obtained for further analysis; (B) 16 DEGs presented non-zero coefficient when log(lambda) equal to -3.846. Figure S2. Mutational analysis of CESC between different risk groups. (A) Top 20 mutated genes in high-risk group; (B) Top 20 mutated genes in low-risk group; (C) Significant mutated genes between high-risk group and low-risk group; (D) Lollipop chart to show the different mutate sites of PDE3A; (E) Co-occurrence and mutually exclusive patterns in high-risk group; (F) Co-occurrence and mutually exclusive patterns in low-risk group. [file 12935_2024_3257_MOESM1_ESM.docx]

**Table S1 siRNA sequence of PRPF40B**

| Gene | Sequence | |
| --- | --- | --- |
|  | Sense（5'-3'） | Antisense（5'-3'） |
| *PRPF40B* siRNA1 | GGCGCAUCUACUACUACAATT | UUGUAGUAGUAGAUGCGCCTT |
| *PRPF40B* siRNA2 | GGGAUGAGUAGUGUCAACUTT | AGUUGACACUACUCAUCCCTT |

**Table S2 Primer sequence of RT-qPCR**

| Gene | Sequence | |
| --- | --- | --- |
|  | Forward（5'-3'） | Reverse（5'-3'） |
| *PRPF40B* | CCACCACTCACACAGATACCA | GGCCACATGCTCACTCCATA |
| *β-Actin* | ACAGAGCCTCGCCTTTGC | CCACCATCACGCCCTGG |

**Table S3 Detailed information of training and testing cohort**

| Clinical parameters | Training cohort  (n = 208) | Testing cohort  (n = 88) | Statistics  value | *P* value |
| --- | --- | --- | --- | --- |
| Age | 48.95 ± 14.44 | 46.08 ± 11.91 | 10122 (w) | 0.150 |
| Grade |  |  | 0.857 (χ^2^) | 0.652 |
| Grade 1-2 | 109 | 41 |  |  |
| Grade 3-4 | 81 | 38 |  |  |
| Grade X | 18 | 9 |  |  |
| Stage |  |  | 2.193 (χ^2^) | 0.334 |
| Stage Ⅰ-Ⅱ | 154 | 72 |  |  |
| Stage Ⅲ-Ⅳ | 49 | 15 |  |  |
| Stage X | 5 | 1 |  |  |
| Survival years | 2.78 ± 2.97 | 3.22 ± 3.48 | 8417.5 (w) | 0.276 |
| Vital status |  |  | 3.910 (χ^2^) | 0.048 |
| Alive | 150 | 73 |  |  |
| Dead | 58 | 15 |  |  |

**Table S4 Univariate Cox result in the training cohort**

| Gene | Hazard ratio | coefficient | *P* value | Lower 95%CI | Upper 95%CI |
| --- | --- | --- | --- | --- | --- |
| HENMT1 | 0.654 | -0.425 | 0.001 | 0.505 | 0.847 |
| SBDS | 1.56 | 0.445 | 0.002 | 1.179 | 2.063 |
| AIMP2 | 1.602 | 0.471 | 0.003 | 1.172 | 2.19 |
| RNASEH2A | 0.72 | -0.328 | 0.004 | 0.575 | 0.903 |
| TYW1 | 1.5 | 0.406 | 0.009 | 1.109 | 2.03 |
| DCP1B | 1.482 | 0.393 | 0.010 | 1.099 | 1.997 |
| GAPDH | 1.423 | 0.353 | 0.010 | 1.089 | 1.861 |
| ZC3HAV1L | 1.498 | 0.404 | 0.010 | 1.101 | 2.04 |
| CCRN4L | 1.398 | 0.335 | 0.014 | 1.069 | 1.829 |
| DDX26B | 0.74 | -0.301 | 0.015 | 0.58 | 0.943 |
| BZW2 | 1.425 | 0.354 | 0.016 | 1.069 | 1.9 |
| RBMS3 | 1.369 | 0.314 | 0.017 | 1.058 | 1.772 |
| ENOX1 | 1.384 | 0.325 | 0.018 | 1.056 | 1.812 |
| WRAP53 | 0.728 | -0.318 | 0.021 | 0.555 | 0.954 |
| ISG20 | 0.725 | -0.322 | 0.022 | 0.55 | 0.955 |
| AFF4 | 1.446 | 0.369 | 0.025 | 1.048 | 1.995 |
| ANGEL2 | 1.346 | 0.297 | 0.030 | 1.029 | 1.761 |
| LRRFIP1 | 1.368 | 0.313 | 0.037 | 1.019 | 1.836 |
| WBP4 | 1.355 | 0.303 | 0.037 | 1.018 | 1.801 |
| RBM38 | 0.761 | -0.273 | 0.038 | 0.588 | 0.984 |
| RBFOX2 | 1.35 | 0.3 | 0.038 | 1.017 | 1.792 |
| MRPS23 | 1.397 | 0.334 | 0.038 | 1.018 | 1.917 |
| PRPF40B | 1.294 | 0.258 | 0.042 | 1.01 | 1.659 |
| CAPRIN2 | 1.311 | 0.27 | 0.047 | 1.004 | 1.711 |
| SNRPN | 0.758 | -0.277 | 0.049 | 0.575 | 1 |
| KHNYN | 1.335 | 0.289 | 0.049 | 1 | 1.782 |


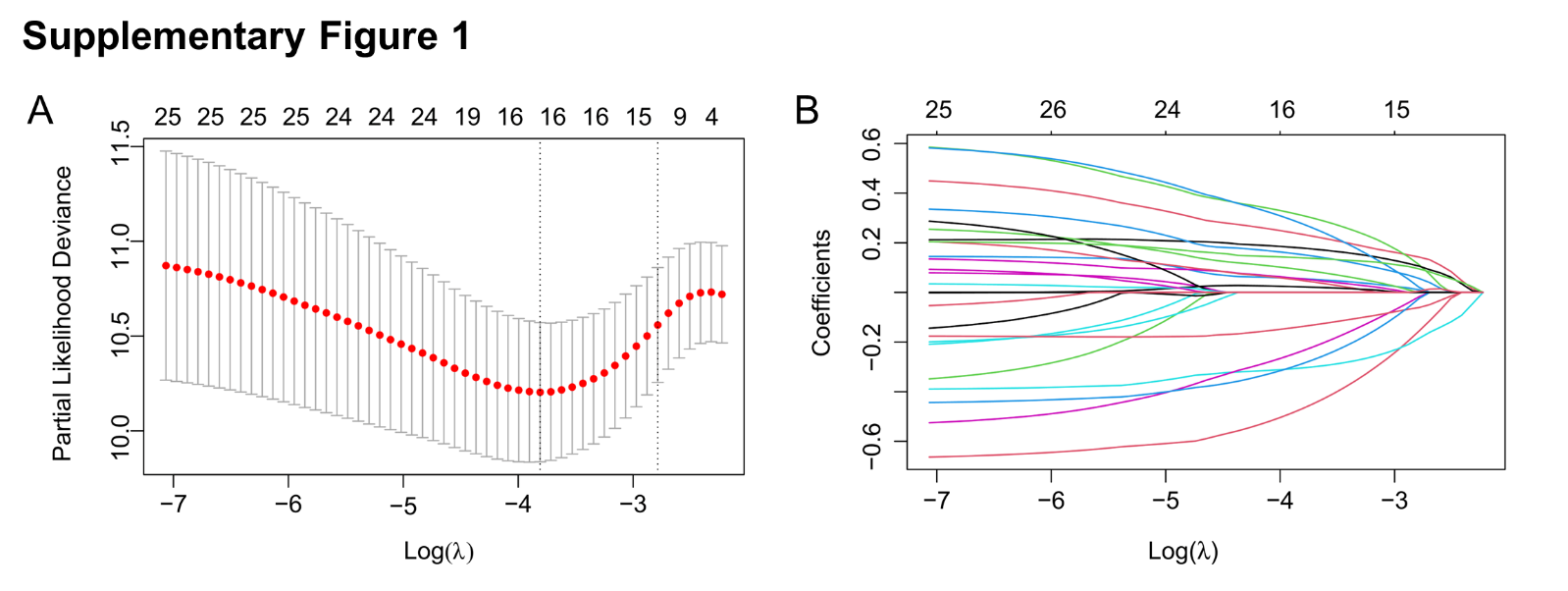
 **Figure S1** The process of lasso cox regression. (**A**) The partial likelihood deviance was minimal when log(lambda) equal to -3.846, and 16 DEGs were obtained for further analysis; (**B**) 16 DEGs presented non-zero coefficient when log(lambda) equal to -3.846.


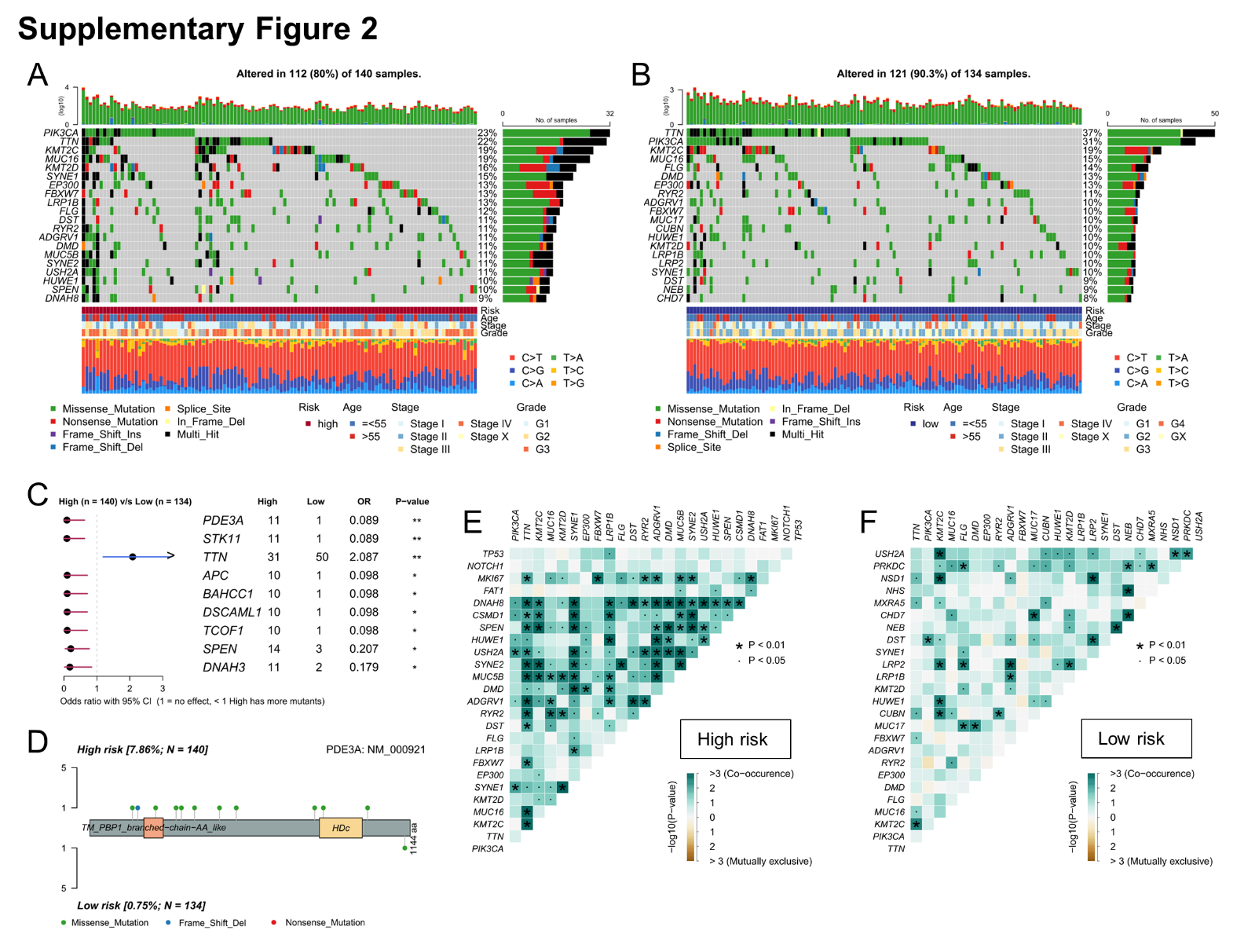
**Figure S2** Mutational analysis of CESC between different risk groups. (**A**) Top 20 mutated genes in high-risk group; (**B**) Top 20 mutated genes in low-risk group; (**C**) Significant mutated genes between high-risk group and low-risk group; (**D**) Lollipop chart to show the different mutate sites of PDE3A; (**E**) Co-occurrence and mutually exclusive patterns in high-risk group; (**F**) Co-occurrence and mutually exclusive patterns in low-risk group.
